# Intensive Care Unit-Acquired Weakness: Not Just Another Muscle Atrophying Condition

**DOI:** 10.3390/ijms21217840

**Published:** 2020-10-22

**Authors:** Heta Lad, Tyler M. Saumur, Margaret S. Herridge, Claudia C. dos Santos, Sunita Mathur, Jane Batt, Penney M. Gilbert

**Affiliations:** 1 Institute of Biomedical Engineering, University of Toronto, Toronto, ON M5S 3G9, Canada; heta.lad@mail.utoronto.ca; 2 Donnelly Centre for Cellular and Biomolecular Research, University of Toronto, Toronto, ON M5S 3E1, Canada; 3 Rehabilitation Sciences Institute, University of Toronto, Toronto, ON M5G 2A2, Canada; tyler.saumur@utoronto.ca; 4 Division of Critical Care Medicine, University Health Network, Toronto, ON M5G 2C4, Canada; margaret.herridge@uhn.ca; 5 Keenan Research Center for Biomedical Science, St. Michael’s Unity Health Toronto, Toronto, ON M5B 1T8, Canada; claudia.dossantos@unityhealth.to; 6 Department of Medicine, University of Toronto, Toronto, ON M5S 3H2, Canada; 7 Department of Physical Therapy, University of Toronto, Toronto, ON M5G 1V7, Canada; sunita.mathur@utoronto.ca; 8 Department of Cell and Systems Biology, University of Toronto, Toronto, ON M5S 3G5, Canada

**Keywords:** muscle atrophy, critical illness, intensive care unit-acquired weakness, critical illness myopathy, critical illness polyneuropathy, COVID-19, SARS-CoV-2, biomarkers

## Abstract

Intensive care unit-acquired weakness (ICUAW) occurs in critically ill patients stemming from the critical illness itself, and results in sustained disability long after the ICU stay. Weakness can be attributed to muscle wasting, impaired contractility, neuropathy, and major pathways associated with muscle protein degradation such as the ubiquitin proteasome system and dysregulated autophagy. Furthermore, it is characterized by the preferential loss of myosin, a distinct feature of the condition. While many risk factors for ICUAW have been identified, effective interventions to offset these changes remain elusive. In addition, our understanding of the mechanisms underlying the long-term, sustained weakness observed in a subset of patients after discharge is minimal. Herein, we discuss the various proposed pathways involved in the pathophysiology of ICUAW, with a focus on the mechanisms underpinning skeletal muscle wasting and impaired contractility, and the animal models used to study them. Furthermore, we will explore the contributions of inflammation, steroid use, and paralysis to the development of ICUAW and how it pertains to those with the corona virus disease of 2019 (COVID-19). We then elaborate on interventions tested as a means to offset these decrements in muscle function that occur as a result of critical illness, and we propose new strategies to explore the molecular mechanisms of ICUAW, including serum-related biomarkers and 3D human skeletal muscle culture models.

## 1. Introduction

Intensive care unit-acquired weakness (ICUAW) can be defined as clinically detected weakness in critically ill patients where the only plausible etiology is the critical illness itself, and which may persist long after ICU discharge [1,2]. By comparison, muscle atrophy, where disuse/unloading is the root cause, almost always resolves with exercise and time [3]. Approximately 40% of critically ill patients experience ICUAW [4]; this is associated with prolonged ICU and hospital stay, duration of mechanical ventilation (MV), and increased ICU and hospital mortality [5,6,7,8,9,10,11].Functional outcomes are heterogenous, while some individuals recover fully to pre-ICU status, others will experience persistent weakness. Sustained weakness results in functional limitations, decreased employment rates, and quality of life [12,13].

Risk factors for ICUAW include sepsis and/or shock, multiple organ failure, metabolic variables such as hyperglycemia, and interventions such as duration of MV [14]. Presently, there is no gold standard for the early diagnosis of ICUAW. Once muscle weakness is evident, physical examination, electromyography, and nerve conduction studies [2] are used to determine the presence of critical illness polyneuropathy (CIP), myopathy (CIM), or a combined critical illness neuromyopathy [2,15,16,17]. The distinction between CIP and CIM depends on electrophysiological or histological evidence of peripheral nerve or muscle fiber dysfunction, respectively. Considerable overlap between the two syndromes makes differentiation difficult. Although CIP and CIM may continue to impact outcomes in survivors of critical care long after ICU discharge, the effects of CIP may be more persistent [18,19,20,21].

Despite increased understanding of risk factors, the etiology of ICUAW remains unclear. Unique features of ICU patients with diseases, such as the coronavirus disease of 2019 (COVID-19), may provide insight into the impact of prolonged sedation, systemic inflammation, and steroid use on the presence of ICUAW [22,23,24]. Improved knowledge, specifically at a molecular level, is critical to the development of future therapeutics aimed at preventing, reducing, and/or enhancing repair of ICUAW. Here, we will review human and animal studies, highlighting the current understanding of the molecular pathogenesis of ICUAW, focusing on the muscle pathology and emphasizing unique characteristics as compared to the atrophy and muscle dysfunction induced by disuse alone. We will further discuss the potential for interventions to target these specific mechanisms and ameliorate muscle dysfunction following an ICU stay. We will also discuss contributions of inflammation and steroid paralysis in the development of ICUAW, as is directly related to recent observations seen in the COVID-19 infected ICU patient population. Lastly, we will examine the knowledge gaps and propose implementation of innovative methods to study critical illness mediated muscle loss and dysfunction in vitro.

## 2. Hallmarks of Muscle Atrophy in the ICU

**Figure 1:**
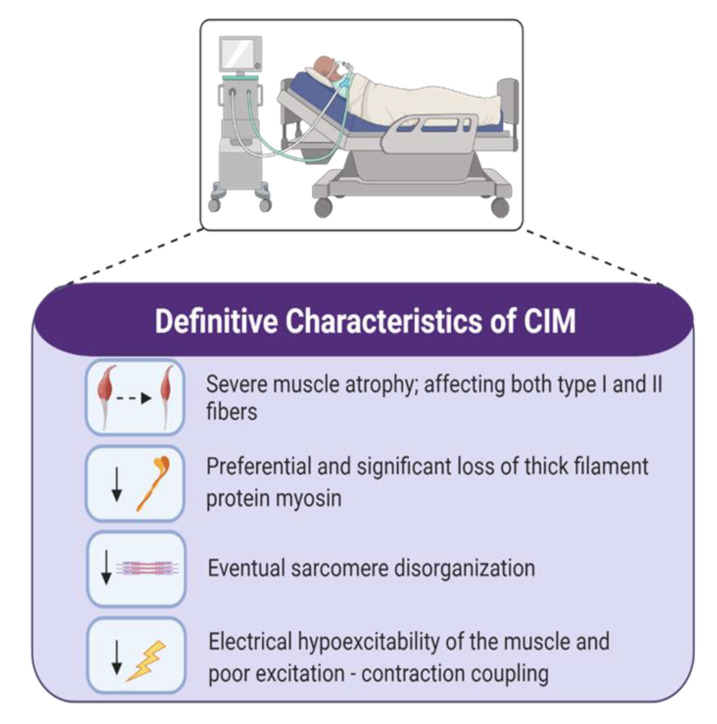
Changes seen in patients with definite critical illness myopathy (CIM) leading to intensive care unit-acquired weakness. CIM is almost exclusively associated with severe atrophy, preferential loss of myosin, and altered muscle cell excitability.

During ICU stay, 25% to 75% of mechanically ventilated, critically ill patients develop severe skeletal muscle atrophy and weakness [2]. Muscle unloading caused by disuse and immobilization may contribute to CIM, however they are not sole causative factors. In the ICU, marked decline in muscle mass can be attributed to changes in muscle fiber cross-sectional area (CSA). Muscle fibers isolated from tibialis anterior and vastus lateralis biopsies from CIM patients exhibit 70% reductions in CSA in fibers expressing the type I myosin heavy chain (MyHC) isoform and 75% reductions in type II MyHC isoform [25]. In comparison, following 6 weeks of bedrest alone, vastus lateralis single fiber analyses showed a more modest 13% decrease in CSA, and a 38% decrease in overall protein content [26] and these changes were not associated with a marked preferential loss of myosin or myosin-associated proteins, as is reported in ICU patients with CIM [27]. Concurrent with the preferential loss of myosin, a reduction in myosin/actin ratio has been observed at a single fiber level in CIM-associated atrophy [25,27,28,29] while there is minimal change in the myosin/actin ratio in different fiber types with bedrest/disuse alone [30,31]. In addition, cross-striation patterning is lost in the majority of ICU patients [27], and is indicative of sarcomere disorganization, and complete sarcomere disruption has been observed in 100% of CIM patients 7 days post-ICU discharge [32]. At a functional level, the preferential loss of myosin has been correlated with a reduction in specific force [25,27], and non-excitable muscle membranes in response to direct muscle stimulation after 1 week in the ICU [33]. A 40% decline in specific tension was also observed at a single fiber level following 6 weeks of bedrest [26]. Thick filament loss is further associated with a change in force generation-associated calcium (Ca^2+^) sensitivity, whereby more Ca^2+^ is required to recruit the number of cross-bridges needed to activate the contractile proteins for force production [25]. Based upon analysis of vastus lateralis biopsies from ICU patients, myofiber CSA was reduced in all fiber types, but was more pronounced in type II-fast twitch fibers [33,34,35]. This may suggest a fiber type switch from fast-to-slow, however further study of fiber type proportions in ICUAW is needed. In contrast, while disproportionate loss of thin actin filaments in the human soleus muscle after 17 days of best rest is reported, in other studies myosin filament concentration appears unchanged [36,37]. Muscle atrophy attributed to disuse is associated with a shift in MyHC isoforms from slow-to-fast as evidenced by a decrease in MyHC slow (type I) and an increase in MyHC fast (type II) following a prolonged period of bedrest [30,38,39], a feature that does not seem to be present in ICUAW. Characteristics of CIM have been outlined in Figure 1.

## 3. Models of ICUAW

The clinical heterogeneity of ICU patients has made it challenging to ascertain ICUAW causality [14,40]. Further, CIM is not the result of a single isolated mechanism [41]; thus, in vivo models that simulate a sequence of events leading to CIM and enable time course analyses are desirable. The availability of such models enables researchers to uncover mediators and mechanisms involved in early versus late phases of ICUAW, and to also test the efficacy of different interventions at variable stages. Additionally, models that can systematically investigate various combinations of risk factors and differentially attribute factors to the distinct features of limb, masticatory and diaphragmatic weakness seen in ICUAW, are key. Below, we briefly review mechanically ventilated animal models as these have successfully reproduced some aspects of ICUAW pathology (summary in Table 1). For excellent reviews of rodent genetic models of ICUAW-associated molecular mediators, see [42,43,44,45].

### 3.1. Porcine Ventilation Models of ICUAW

The ability of porcine models to sustain MV for days has permitted simulation of ICUAW triggered by various mechanisms and manifesting in the development of electrophysiological phenotypes similar to those observed in CIM patients. Porcine models are advantageous in the study of disease as they are comparable to humans in terms of many physiological and anatomical structures, and disease pathogenesis [59]. The pig has been used to evaluate prolonged MV (5 days) on diaphragmatic activity, and confirmed the negative effects induced by diaphragm muscle unloading after 5 days of MV [60]. Similarly, ventilated, immobilized, and deeply sedated pigs, either in isolation, or in the presence of corticosteroids, neuromuscular blocking agents (NMBAs), and/or sepsis, have been used to model ICUAW [47,50]. Piglets subjected to 5 days of MV presented with minimal change in muscle fiber CSA [46,47,48] or specific force in limb muscles [46,47], but a decrease in specific force was observed when ventilation was administered in combination with sepsis, corticosteroids, or all three (steroids, sepsis, and NMBAs) [47,48,49,50]. While preferential loss of myosin, and major alterations in the myosin/actin ratio was not highly significant [47,48], transcriptional downregulation of sarcomeric proteins belonging to both the thick and thin filaments was observed on day 5 of ICU intervention, concurrent with an upregulation of the ubiquitin proteasome system [46]. Absence of muscle fiber atrophy despite increased proteolysis and decreased expression of sarcomeric proteins may be attributed to the half-life of the thin and thick filament proteins being longer than the 5 days of ICU intervention that was implemented in this porcine model [61].

While the histological evidence of myopathy seen in human patient populations was not recapitulated in this porcine model, the characteristic electrophysiological abnormalities were observed. Compound muscle action potential (CMAP) decreased by 50% in all groups in this study, irrespective of the intervention received by animals (NMBAs, corticosteroids, sepsis) [47], and others have reported a 97% decrease in CMAP from days 1–5 of ICU intervention [48]. Similarly, sepsis-induced myopathy in the context of a porcine ICU model study was associated with muscle membrane dysfunction as early as 6 h after sepsis was induced [51]. In a follow-up to this study, animals with high mean arterial pressure had an extended relative refractory period and lower early [53]. This suggests that high mean arterial pressure may correlate with muscle membrane electrophysiology, perhaps owing to issues with perfusion; and in keeping with this observation, muscle dysfunction positively correlated with norepinephrine dose [53].

Using the porcine model, differences in the response of specific muscle groups in the absence or presence of NMBAs, corticosteroids, and sepsis were apparent, and possible mechanisms reported. For example, while limb and respiratory muscles exhibited a 45% reduction in their specific force, masticatory muscle showed no decline [47,49]. Increased expression levels of oxidative stress response genes, protein chaperones, and growth promoting factors in masticatory muscles were proposed to protect from, or compensate for the muscle atrophic stimuli [49]. Compared to hindlimb muscles, severe ventilator-induced diaphragmatic dysfunction was speculated to arise from sarcoplasmic reticulum ryanodine receptor remodeling and the abnormal sarcoplasmic reticulum Ca^2+^ leakage at rest observed in the mechanically ventilated animals [52].

Despite their advantages, current porcine ventilation model studies have failed to capture distinguishing molecular hallmarks of ICUAW. This may be in part due to the stage of development at which porcine models are often studied. Typically, pediatric pigs weighing less than 30 kg are used [48]. Therefore, the anabolic drive of a growing pig may interfere with the ability to study catabolic changes, although, it would arguably be more difficult to use large adult (100–120 kg) pigs in these long-term studies. Additionally, studies using porcine models are conducted in relatively small sample size, thereby limiting statistical power. Significant cost and ethical consideration also tend to limit the use of the model.

### 3.2. Rat Ventilation Models of ICUAW

Rodents are more cost-friendly than pigs and have been shown to recapitulate specific features of ICUAW experimentally such as muscle membrane hypoexcitability in response to direct stimulation. In a rat model of steroid-denervation, the denervation alone did not significantly alter CMAP. It was the additive effect of systemic steroid administration that resulted in significant reduction in mean CMAP in the *tibialis anterior* muscles, and loss of membrane excitability, possibly owing to a decline in the number of adult isoform sodium channels [62]. Furthermore, the preferential loss of myosin and downregulation of myosin-synthesizing mRNA have been reported in this model [63], albeit the myosin loss was restricted to slow-type fibers. Although the steroid-denervation model recapitulates many pathologic changes that are observed in patients with CIM, these rats are not critically ill, mechanically ventilated, nor limited in weight bearing activity—all dominating triggers promoting muscle atrophy and weakness in the critically ill patient [56]. Moreover, while temporary denervation results from CIP, peripheral nerve denervation is not a characteristic feature of CIM [64]. Ultimately, while the rat steroid-denervation model replicates some pathologic and physiologic hallmarks of ICUAW, underlying cellular mechanisms may be different, and other models for study are therefore required.

A rat model generated by Dworkin and colleagues, originally established to study blood pressure regulation, mimics aspects of the ICU environment by applying isoflurane anesthesia, NMBA-induced paralysis with prolonged MV, and muscle unloading [65]. Though not the intended goal of this model, it recreates key factors that drive the manifestation of ICUAW. Adaptions of this model [65] by modifying sedation, paralysis and MV parameters permitted time resolved analyses (ranging from a few hours to 2 weeks) [56]. After 5 days of the “ICU intervention” a preferential loss of myosin was observed and identified in both slow and fast twitch muscles, and a significant decrease in actin and myosin transcriptional synthesis was revealed [56]. Importantly, and reminiscent of CIM, actin content remained stable throughout all timepoints [56]. These observations agree with clinical CIM data, and contrasts with the rat steroid-denervation model, where thick filament loss was restricted to the slow twitch (soleus) muscles and was not accompanied by a similar reduction in myosin mRNA expression [57]. The fact that preferential loss of myosin was only seen beyond 5 days of MV might explain why myosin loss was not observed in the pig model [47,48,49].

Similar to the porcine model, the rat “ICU” model has enabled characterization and mechanistic study of the differential features inherent to disparate muscle groups in critical illness. For example, loss of specific force was reported to be more pronounced in the diaphragm muscles than what they observed in the limb muscles, and this occurred in the absence of preferential myosin loss seen in the peripheral musculature [54,56].

Importantly, the rat ICU model is able to generate a CIM like picture, even in the absence of corticosteroids and sepsis. This provides the researcher the benefit of being able to investigate the impact of other factors, including for example nutrition or passive muscle loading, as well as sepsis and corticosteroids, either independently or synergistically, on the induction of muscle wasting and weakness “in the ICU” [58,66]. However, the requirement for continual heavy sedation and paralysis while ventilated, and 24 h/day observation and care, limits the practical application of these animal models owing to cost and labor requirements. In addition, continual heavy sedation and use of NMBAs during the entire duration of MV occurs only in a subset of ICU patients; therefore, the absolute recreation of the ICU environment remains elusive.

## 4. Mechanisms and the Potential for Intervention during and after ICU Stay

**Figure 2:**
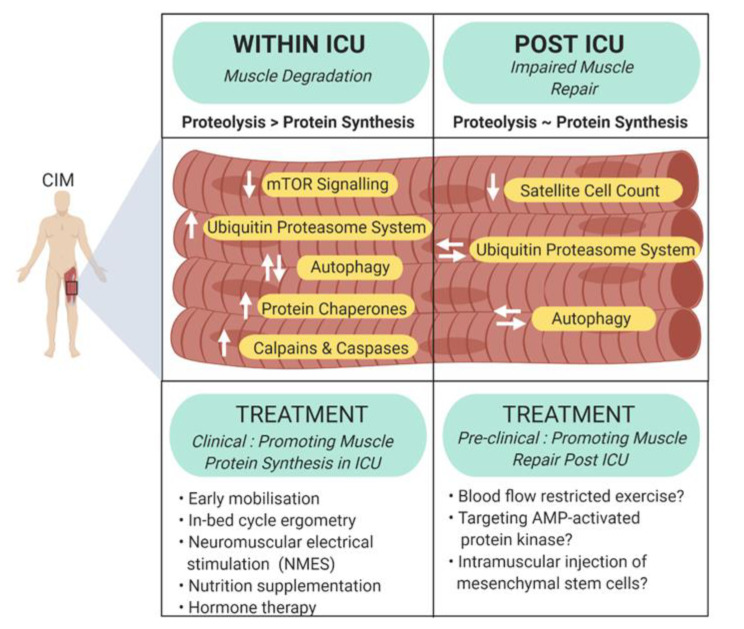
Summary of known molecular mediators of muscle wasting in critical illness. Muscle atrophy is a major myogenic component of critical illness myopathy. Concurrent with intensive care unit stay, muscle degradation occurs due to proteolytic activity outweighing protein synthesis, whereas impaired muscle repair contributes to sustained weakness following intensive care unit discharge. Double vertical arrows represent dysregulation and double horizontal arrows indicate stabilization.

ICUAW is associated with increased ICU length of stay and mortality, increased morbidity and long-term physical disability after hospital discharge [67]. Currently there are no interventions that can consistently prevent muscle loss during critical illness, or reverse the muscle wasting following ICU discharge [68]. Below, we discuss the different mechanisms that may be actively promoting muscle wasting during and after ICU stay, and the recent interventions that are being trialed to combat ICUAW, with a primary focus on human studies (Figure 2).

### 4.1. Mechanisms of Muscle Catabolism during ICU Stay

During muscle catabolism, the rate of protein degradation exceeds the rate of protein synthesis, driving a net catabolic state [69]. In critical illness, muscle protein synthesis may be variable, but does not affect muscle to the extent of proteolysis [70,71]. Muscle protein degradation is rapidly enhanced with patients losing as much as 20% of their muscle mass in the first 10 days of ICU stay [71], suggesting that muscle proteolysis may be the key driver of muscle wasting in the ICU. Recent work has shown that this increase in muscle protein degradation is attenuated during the later stages of ICU stay [72]; however, the negative impact on muscle functionality and quality of life can persist years after ICU discharge [2]. Two major pathways that play a critical role in facilitating muscle protein degradation are the ubiquitin proteasome system (UPS) and dysregulated autophagy.

#### 4.1.1. The Ubiquitin Proteasome System in Critical Illness

In brief, the UPS is an adenosine triphosphate (ATP)-dependent proteolytic pathway that enables the degradation of target proteins [73,74]. These target proteins are tagged with ubiquitin (Ub) molecules by the E3 Ub-protein ligase, enabling the large protease complex—26S proteasome—to degrade identified protein targets into smaller peptides [73,74]. A more in-depth review of the UPS can be found here [75]. Two key E3 Ub-protein ligases, muscle-atrophy f box protein (MAFbx)—also called atrogin-1—and muscle ring finger protein 1 (MuRF1), are involved in muscle atrophy through their increased activity [76]. However, others including MuRF2, FBOX31, SMART, and TRIM 32, may also play a role [74]. MAFbx and MuRF1 are elevated in numerous models of muscle atrophy including immobilized rats [77], diabetes mellitus [73], denervation [77], sepsis [78], amongst many more. Consistently, in knockout mice deficient in MAFbx or MuRF1, muscle atrophy is minimal compared to control mice, with knockout mice resistant to denervation-induced atrophy [77]; additionally, the overexpression of MAFbx in myotubes was shown to produce severe atrophy [77].

Many triggers common to ICU patients can stimulate activity of the UPS [41]. Fractions containing cytosolic soluble and membrane-bound proteasomes generated from leg muscle samples of ICU patients on ventilator support for at least one week revealed a 30% elevation in the proteolytic activity of membrane-bound proteases, however this increase was not observed in the soluble proteasomes [79]. In a follow-up study with septic ICU patients, a 45–55% increase in proteasome activity in the leg muscles compared to healthy references was reported [80]. Interestingly, respiratory muscles behaved in a similar fashion, with proteasome activity 30% higher in patients with sepsis compared to controls [80]. In accordance, the serum of septic and critically ill trauma patients possessed significantly elevated 20S proteasome activity (a subunit of the 26S proteasome) [81]. Vastus lateralis biopsies from critically ill patients also exhibit an elevation in 20S proteasome, MuRF1 and MAFbx mRNA and protein expression [82]. While others have also shown similar findings [70,79,83], the literature is not consistent and lack of differential expression of the ubiquitin ligases in patients afflicted with CIM has also been reported [84]. The reason for these discrepancies is not clear but may relate to the timing of biopsy collection, muscle group sampled, and analysis of transcript vs. protein levels. Despite the role the UPS plays in the rapid muscle wasting experienced in the ICU, patients experiencing sustained muscle atrophy following critical illness resolution displayed UPS activity comparable to healthy controls at 6 months post-ICU discharge [32].

Inhibition of UPS may be a therapeutic option to prevent ICUAW. Proper timing of proteasome inhibition, however, will be crucial, since its use in pre-clinical models is associated with delay and attenuation of resting energy expenditure as well as increased mortality, specifically when implemented early after injury [85].

#### 4.1.2. Dysregulated Autophagy in Critical Illness

Dysregulation of autophagy may play a role in skeletal muscle atrophy. Autophagy is a conserved intracellular degradation process essential to the maintenance of normal cell physiology in response to different forms of stress [86,87,88]. Autophagy can be selective or non-selective; initiated by de novo synthesis of phagophores [86,87,88]. Phagophores engulf intracellular cargo, and form double-membraned autophagosomes which ultimately fuse with lysosomes to form autolysosomes [86,87,88]. In the autolysosomes, the autophagic cargo is degraded by lysosomal hydrolases. The resulting by-products of degradation are returned to the cytoplasm where they can be reused [86,87,88]. In skeletal muscle, both heightened and diminished autophagy will lead to muscle loss and weakness [89]. In *Atg7* deficient mice, absence of this critical autophagy gene results in myofiber degeneration and a build-up of abnormal intracellular mitochondria, muscle weakness and wasting [90,91]. Under conditions of denervation and fasting, the inhibition of autophagy exacerbates muscle loss, suggesting that autophagy may be crucial for myofiber maintenance [90,91]. In other genetic mouse models, upregulation of autophagy is associated with muscle wasting [92]. In critically ill patients, rectus abdominis biopsies revealed increased levels of positive regulators of autophagy (beclin-1 and the Atg5-Atg12 complex), as well as decrease in mature autophagic vesicles indicated by an increase of LC3-I, but not LC3 -II, which is a readout for mature autophagosome formation [93]. Accumulation of p62 suggests autophagy is inhibited in these patients [93]. Transcriptional upregulation in genes that facilitate autophagy has been noted in leg muscle biopsies [84] and autophagy has been shown to be upregulated in the vastus lateralis of critically ill patients mechanically ventilated for one week when compared to healthy controls. However, by 6 months post-ICU discharge, there was no difference in the activity of most autophagy markers [32]. In summary, balanced autophagy is necessary to maintain normal turnover in skeletal muscle, and like the UPS, therapeutic interventions to attenuate dysregulated autophagy should be trialed at earlier stages of ICUAW.

#### 4.1.3. Mitochondrial Dysfunction in the ICU

A vital role of mitochondria is to supply ATP so that cellular energy demand is met. This is primarily accomplished by oxidative phosphorylation (aerobic respiration) [94]. During critical illness, and the onset of CIM, mitochondrial dysfunction is present [95,96,97,98,99]. Impaired mitochondrial function was found in limb muscle biopsies of critically ill patients, indicated by reduced activity levels of respiratory chain enzymes such as cytochrome c oxidase and succinate dehydrogenase [95,96,99]. Moreover, in critical illness non-survivors, citrate synthase activity was 40% lower in limb muscle biopsies [96]. Similarly, citrate synthase was ~50% lower in the intercostal muscles of ICU patients alongside a decrease in mitochondrial enzyme activity [97], suggesting similar mitochondrial dysfunction between respiratory and limb muscles. Moreover, several genes encoding proteins that promote mitochondrial biogenesis (e.g., PGC-1α, TFAM) and mitochondrial dynamics were downregulated [95,96,98]. Interestingly, in survivors of critical illness, the reduction in mitochondrial respiratory transcripts and protein levels were impeded by the early activation of oxidative stress response and mitochondrial biogenesis [96]. Additionally, critically ill patients were also found to have a lower mitochondrial density [32,97], which was reported to normalize by 6 months post ICU discharge [32]. Finally, oxidative phosphorylation was significantly impaired, indicated by the marked reduction in ATP synthesis in muscle biopsies from critically ill patients [97,98,99]. Thus, it is evident that energy provision is compromised in critically ill patients, which can negatively impact the feasibility of usual interventions such as the early mobilization strategies used to promote muscle protein synthesis within the ICU. However, this may be different for critical illness survivors who have been discharged from the ICU.

#### 4.1.4. Cytokine Elevation in Critical Illness

Meta-analysis of systemic inflammation and muscle strength and/or muscle mass has revealed that higher levels of circulating inflammatory markers are associated with a marked decrease in skeletal muscle strength and mass [100]. This has been increasingly noticeable in the severe acute respiratory syndrome coronavirus 2 (SARS-CoV-2) infected patient population, with many reports of virally driven hyperinflammation [22,23,101]. Many similarities between CIM and hospitalized COVID-19 patients in the ICU have been reported. These include prolonged ventilation and administration of other ICU interventions, the presence of myalgia, significantly reduced and prolonged duration of CMAP, and significant muscle loss depicted by anorexia in COVID-19 patients [24,102,103]. Another commonality between COVID-19 patients and those with ICUAW are the reported cytokines involved in hyperinflammation discussed below. A subset of proinflammatory cytokines are suggested to stimulate muscle atrophy and weakness during critical illness [104]. Of these, three cytokines, tumor necrosis factor alpha (TNFα), interleukin 1 (IL-1), and interleukin 6 (IL-6) are the most well investigated in critically ill patients [41]. Early studies showed that incubation of cultured myotubes with clinically relevant doses of TNFα caused progressive reduction in myotube diameter and total muscle protein content [105,106]. Blood collection over consecutive days within the ICU, and analysis of serum cytokine levels, revealed consistently elevated TNFα levels probing for further investigation of this cytokine [107]. In fact, others revealed that maximal plasma levels of TNFα were higher in patients who developed ICUAW, than control subjects [108]. There is also evidence of elevated TNFα present in blood and diseased tissues of patients with COVID-19 [109], and TNFα levels during time of hospitalization was an independent predictor of patient survival, disease severity, and death [110]. TNFα also activates the mitogen-activated protein kinases, and this signaling upregulates MuRF1 and atrogen-1 facilitating UPS mediated degradation in the skeletal muscle [78]. Moreover, IL-1 is also a potential cytokine driving muscle atrophy seen in critically ill patients. IL-1 infusion for 6 days reduces muscle weight and protein content of the rat gastrocnemius [111], and administration of IL-1 antagonist can preserve muscle mass in a septic rat model [112]. Similarly, a phase 3 randomized controlled trial of the IL-1 blockade, anakinra, showed significant survival benefits in patients with sepsis [113]. The incubation of skinned muscle fibers with exogenously applied IL-1α revealed colocalization of IL-1α with ryanodine receptor 1, which completely and reversibly inhibiting sarcoplasmic Ca^2+^ release in skeletal muscle, possibly contributing to muscle weakness [114]. IL-1 is also highly elevated in the plasma of COVID-19 patients, with severe infection requiring ICU admission [115]. Finally, the role of IL-6 has also been investigated in critical illness. IL-6 production is stimulated by TNFα, and IL-1β, and may contribute to the systemic inflammation in critical illness and sepsis [116]. Myotubes treated with plasma from septic shock patients had a marked increase in levels of IL-6 compared to control samples, concurrent with significantly decreased myosin content and upregulated activity of MuRF1 and atrogen-1 in the skeletal muscle cultures [117]. Interesting, the addition of exogenous IL-6 to control cultures did not induce a phenotype similar to that seen with plasma from septic shock patients, suggesting an indirect method of action by IL-6 in vivo [117]. Interestingly, predictors of fatality from a recent retrospective multicenter study of 150 COVID-19 cases included blood levels of IL-6, and suggested that mortality may be due to hyperinflammation driven by the viral infection [118]. Others have also reported IL-6 serum levels to be an independent and significant predictor of disease severity and death [110,119]. A multicenter randomized controlled trial of IL-6 receptor blockade, tocilizumab, has been approved for administration in China for patients with COVID-19 pneumonia and elevated IL-6 levels [120]. Ultimately, these reports suggest a role of cytokine elevation in skeletal muscle atrophy in events of systemic inflammation, such as those seen during sepsis and COVID-19 infection. Specifically, during the first few days of ICU stay, systemic inflammation is increased in patients who developed ICUAW compared to those patients who do not [121] as well as those with a more severe COVID-19 infection and thus higher risk of fatality [110].

#### 4.1.5. Other Mechanisms Contributing to Muscle Loss and Weakness: Calpains, Caspases, and Chaperones

Mechanisms behind muscle protein degradation are not limited to upregulated UPS or dysregulated autophagy. Proteolytic caspase family members have been shown to be variably upregulated in animal models of critical illness and sepsis [41]. Similar behaviors are also observed in proteolytic calpains who, like caspases, may participate in the degradation of actomyosin complexes for subsequent degradation by the UPS [41]. Additionally, RNA-sequencing analyses of patients with CIM revealed a change in calpain expression [84].

Chaperone proteins—including heat shock protein (HSP) 70 and *α*B-crystalline—were upregulated in the first 5 days in animal models obtaining ICU treatments [122,123]. The response of the chaperones may be to protect the skeletal muscle from protein loss, which ultimately fail, as critical illness progresses, and proteolysis continues. This supposition was supported in a more recent study in rats, where treatment with chaperone co-inducer *BGP-15* improved soleus muscle fiber function during the early stages of ICU exposure, but was ineffective after the manifestation of preferential loss of myosin [124]. Additionally, RNA-sequencing data of CIM patients revealed that the myosin chaperone *UNC-45B*, regulating myosin folding, assembly, and function, was upregulated [84]. Therefore, protein chaperones may be a compensatory mechanism, regulated early to protect muscle structural proteins from proteolysis.

#### 4.1.6. Potential Treatments

The enhanced proteolytic mechanisms delineated active within the ICU attenuate at stages where ICUAW has manifested and muscle atrophy is sustained after ICU discharge. Therefore, the window of opportunity for therapeutic interventions targeting these mechanisms is early during the course of CIM. For mechanisms that are dysregulated, such as autophagy, appropriate timing, extent of activity, and inhibition all need to be considered to regulate autophagy during the appropriate window before ICUAW is established. Metformin is a commonly used drug in the treatment of type 2 diabetes [125], and has received a lot of attention for use in anti-cancer therapy [126,127]. Metformin induces autophagy by activating AMP-activated protein kinase (AMPK) and inhibiting the mammalian target of rapamycin (mTOR) [126,128,129]. In diabetic patients, a 10 week metformin treatment period markedly increased skeletal muscle AMPK activity [130]. In a murine burn model, treatment with metformin activated AMPK levels in skeletal muscle and mitigated muscle wasting such that the muscle CSA 7 days following burn injury was comparable to control mice [131]. In this study metformin treatment also resulted in an increase of cells expressing the muscle stem cell marker Pax7+, 7 days post-burn injury in conjunction with an increase in total Pax7 protein levels [131]. Thus, metformin may be a potential treatment option to induce autophagy and attenuate skeletal muscle wasting as occurs in the development of ICUAW. Other autophagy activators such as rapamycin, everolimus, perifosine, and resveratrol may also be of interest [127]. Still, autophagy is considered a double-edged sword, and studies to optimize the timing of autophagy inhibition are very important to maintain normal turnover in skeletal muscle. Moreover, through activation of AMPK, metformin has also been shown to increase mitochondrial respiration, and ATP levels in mouse hepatocytes [132]. These data suggest that metformin may be beneficial in targeting both dysregulated autophagy and mitochondrial function, however the investigation of skeletal muscle under conditions of critical illness are needed to deem its efficacy. Alternatively, mitochondrial biogenesis can also be stimulated through peroxisome proliferator-activated receptor agonists such as bezafibrate [133,134]. Treatment of fibroblasts or myoblasts with bezafibrate from patients with deficient mitochondrial respiratory chain activity resulted in a stimulatory effect on respiratory chain complexes at both the mRNA and protein levels [135]. Therefore, pharmacological activation of mitochondrial biogenesis may be beneficial in targeting dysregulation mitochondrial function in ICU, especially in conjunction with energy demanding interventions such as early mobilization, and warranting further investigation.

Similar considerations apply for the UPS. Bortezomib is the first proteasome inhibitor approved by the Food and Drug Administration (FDA) for clinical use in the treatment of multiple myeloma that works by inhibiting selective proteasomes and NF-kB signaling [136,137]. In animal models of muscle wasting where the proteasome is hyperactive (e.g., denervation atrophy, Duchenne muscular dystrophy), this compound has also been reported as an effective inhibitor of muscle atrophy [136]. However, the effects of this drug are not universal for all muscle atrophying conditions. Bortezomib has been shown to be ineffective for cancer cachexia [136], and a recent study indicated that multiple myeloma patients treated with this drug experienced metabolic myopathy [137]. Alternative proteasome inhibitors such as carfilzomib, ixazomib, and oprozomib have been generated for the treatment of multiple myeloma, however their specific role in treatment of muscle atrophy is unknown.

Lastly, corticosteroids are commonly administered during critical illness and ICU stay. Glucocorticoids work, in part, by inhibiting NF-kB signaling, which in turn inhibits the synthesis of target genes, including IL-1 and IL-6 [138,139]. However, despite its anti-inflammatory effects, the efficacy of glucocorticoid therapy is controversial. A meta-analysis revealed a significant association between corticosteroid administration and ICUAW, while others have reported increased length of ICU stay and MV with corticosteroid use [140,141]. Similarly, the use of glucocorticoids for the treatment of COVID-19 remains controversial. Meta-analysis revealed that critical patients with severe infection are more likely to require the use of corticosteroids, however, it is associated with higher patient mortality [142]. Thus, antagonists that directly target pro-inflammatory cytokines may be a more desired treatment option to target hyperinflammation in critically ill, and COVID-19 patients.

### 4.2. Decrease in Muscle Synthesis during ICU Stay and Strategies to Counteract It

The homeostatic regulation between protein synthesis and protein degradation in the muscle is lost during critical illness as proteolytic pathways become upregulated. There is increasing interest to develop interventions that accelerate muscle protein synthesis to counteract this net catabolic state. The mTOR signaling network is a key driver of skeletal muscle hypertrophy, by promoting muscle protein synthesis [69,143,144]. mTOR occurs in two complexes, mTORC1 and mTORC2, where rapamycin sensitive complex—mTORC1—is a critical complex in controlling skeletal muscle mass by integrating both extracellular and intracellular signals. mTORC1 increases translational efficiency and promotes muscle protein synthesis primarily by activation of p70S6 kinase 1 and inhibition of eIF4E-binding protein 1, through phosphorylation of these two key effectors [69,143,144]. The activity of mTORC1 can be activated through the canonical IGF-1/AKT/mTOR signaling or in an AKT-independent, mTOR-dependent manner through mechanical or nutritional stimuli [69,143,144]. Loss of these stimuli—through bed rest, and muscle disuse—reduces protein synthesis through the downregulation of these key signaling pathways [55].

In addition to mechanical and nutritional stimuli loss and anabolic resistance, the inability of skeletal muscle to respond to anabolic stimuli (e.g., muscle contraction, hormone stimulation, protein provision) so as to maintain muscle mass, may also occur in critically ill patients [145,146]. Early studies have shown that this suppression of anabolic signaling pathways is simultaneous with the upregulation of muscle proteolytic pathways in ICU patients [82]. More recently, researchers investigating the skeletal muscle metabolic phenotype during early critical illness (within the first week) found that the upregulation of hypoxic and intramuscular inflammatory signaling is heavily, and directly, associated with impaired anabolic signaling [98]. There are no interventions that can consistently prevent and/or treat ICUAW, therefore most interventions focus on eliminating or reducing risk factors. This section will briefly discuss the different strategies that are being tested in the ICU to combat the net catabolic state.

#### 4.2.1. Minimizing Sedation and Implementing Mobilization Strategies in the ICU

Mobilization of critically ill patients is intended to encourage muscle loading and shorten the continuous disuse/immobilization experienced in the ICU, in order to both stimulate muscle protein synthesis pathways and inhibit catabolism. Such interventions require a policy to minimize sedation, since an important barrier to early mobilization is heavy sedation [147,148]. Ceasing sedative infusions early on in the ICU stay is safe and feasible and may be beneficial in decreasing the duration of MV and length of ICU stay [149,150,151], however there is still no evidence that sedatives have a direct impact on ICUAW [147]. Interestingly, critically ill patients with COVID-19 requiring prolonged sedation, do have a higher frequency of ICUAW [24]. Furthermore, animals treated with propofol, a common sedative administered in the ICU, had significant increases in plasma creatine kinase (CK), indicative of muscle damage [152]. Thus, minimizing sedation may be beneficial, and understanding contexts where this benefit is maximized will be important. Moreover, while early mobilization may appear promising [153], the evidence supporting mobilization to improve function in those with ICUAW remains poor [154]. Short-term physical outcomes appear to be more positively impacted by mobilization compared to long-term outcomes. Mobilization has been shown to increase Medical Research Council (MRC) score, decrease the incidence of ICUAW, and improve mobility compared to standard of care or no early rehabilitation [155,156,157]. However, results have varied and larger randomized control trials are needed to further understand the impact of mobilization on physical function. Furthermore, the timing of mobilization may also be important. Early during ICUAW, patients may not have the capacity to generate sufficient energy to actively participate in exercise due to altered mitochondrial function [158]. Therefore, future work focusing on the effect of mobilization throughout the time course of the ICU stay is important. Additionally, there are many other barriers in implementing early mobilization including, but not limited to, delirium or coma, hemodynamic instability, and lack of personnel or equipment [159]. Other physical rehabilitation strategies such as neuromuscular electrical stimulation [160], in-bed cycle ergometry [161], and a combination of the two [162], have shown promising results and are gaining research interest [163]. However, confirmation in larger trials is warranted to confirm these findings in bigger cohorts of patients, and on the effects of these interventions on muscle function and ICUAW-related outcomes in both the short and long term.

#### 4.2.2. Nutritional Strategies

There is evidence that malnutrition in the ICU is associated with poorer patient outcomes [164], and many randomized control trials have been conducted to assess the best timing and the appropriate route of nutrient administration. However, results are inconsistent. Clinical guidelines have recommended higher amino acid/protein provision in critical illness [165,166]. High protein intake—relative to standard—has been reported to be associated with small improvements in various measures (e.g., grip strength); however, length of stay and mortality measures between the two were comparable [167]. Conversely, increased protein during the first week of ICU stay is associated with accentuated muscle wasting; thus, timing of nutritional supplementation also appears to impact outcomes [71]. Critically ill patients administered parenteral nutrition “late” (after day 8) during ICU stay [168] experienced more rapid recovery and fewer complications (e.g., infection and cholestasis), compared to early parenteral nutrition (within 48 h). ICU mortality and 90-day survival rates, however, were similar between both groups [168]. Research has similarly demonstrated muscle weakness experienced by ICU patients resolves faster in those receiving late parenteral nutrition [169].

Meta-analyses of randomized trial data suggest that glutamine supplementation in the ICU may improve patient recovery [170], although the literature remains conflicted [171]. In a large randomized trial, mechanically ventilated patients with multiorgan failure were given supplements of glutamine, antioxidants, both, or placebo within 24 h after ICU admission [171]. Those receiving glutamine supplementation had significantly higher hospital mortality and mortality after 6 months, compared to those who received placebo [171].

Part of the reason for these apparent contradictory results across nutritional studies in the ICU lies in the fact that baseline nutritional and energy requirements vary dramatically with activity level and there is heterogeneity in energy requirements within the critically ill population, which are further amplified if early mobility is prescribed. Few studies have adequately addressed the basal energy requirements or activity level of the patient, within nutritional studies. How, when, and what should be fed to the critically ill patient to improve muscle mass and function in the short and long term remains to be determined.

#### 4.2.3. Hormone Stimulation

Anabolic androgenic steroids, and other hormones such as growth hormone and IGF1, play a key role in muscle protein turnover by specifically stimulating anabolic pathways to promote muscle hypertrophy [172]. Treatment options with these hormones have been explored to combat ICUAW. Hyperglycemia is a critical risk factor of ICUAW, and is associated with an increased risk of morbidity and mortality. Numerous studies have provided evidence that critically ill patients receiving intensive insulin therapy have a decreased likelihood of developing CIP and CIM [173] and reduced morbidity [174,175] and mortality [175]. In contrast, intensive glucose control has been reported to increase the incidence of hypoglycemia and mortality in adult ICU patients offering no significant benefit in terms of length of ICU stay and days of MV compared to conventional glucose control [176]. Insulin stimulates signaling via the AKT/PI3K pathway to induce both protein synthesis and simultaneously downregulate UPS mediated proteolysis and autophagy. Following intensive insulin infusion, CSA of the rectus abdominis and vastus lateralis was smaller and proteolytic enzyme activity was increased compared to conventional glucose management. Loss of thick filament was noted regardless of insulin treatment [29]. The authors argue that results may have been confounded by corticosteroid co-administration [29]. Additional studies have indicated that hyperglycemia in CIM patients is a result of insufficient translocation of glucose transporter-4 to the sarcolemma [177], which is not ameliorated by intensive insulin therapy [29]. Therefore, intensive insulin therapy may not be effective in treating ICUAW-related myopathy, but may attenuate neuropathic features. In brain-injured ICU patients, intensive insulin treatment protects peripheral and central nervous system improving outcomes [178]. Overall, intensive insulin therapy has led to variables outcomes in ICUAW worthy of further exploration. It should be noted however that the risk of hypoglycemia following intensive insulin therapy can have further complications and glucose levels should be monitored closely.

Muscle protein catabolism during critical illness is in part due to the acquired resistance to growth hormone [179]. A randomized controlled trial where ICU patients received either growth hormone or placebo for a maximum of 21 days, revealed that high doses of growth hormone was associated with increased morbidity and mortality [180]. Additionally, MV time and ICU and hospital length of stay were much longer in the growth hormone group [180]. Lastly, low testosterone levels, or hypotestosteronemia, are frequently present in critically ill male patients, and may contribute to longer ICU stay [181,182,183]. As testosterone signals via the mTOR pathway and androgen receptors leading to skeletal muscle hypertrophy [184], hypotestosteronemia may contribute to a net catabolic state resulting in ICUAW. In a septic rat model, short term treatment with testosterone propionate significantly improved maximum contractile force, and CSA of extensor digitorum longus muscle fibers without onset of testicular atrophy [185]. Importantly, testosterone treatment significantly increased protein levels of fast MyHC suggesting that testosterone may be a beneficial treatment to ameliorate ICUAW [185]. Overall, hormone therapy for ICUAW has been met with encouraging pre-clinical data but limited clinical success. Although theoretically possible, further studies are required to address the discrepancy in results.

### 4.3. Muscle Repair and Regeneration after ICU Discharge

While muscle catabolism is one of the primary processes responsible for muscle wasting in the ICU, markers of catabolism have been reported to have normalized at 6 months post-discharge [32]. Quadriceps CSA, UPS-mediated proteolytic and autophagic markers, and mitochondrial content were either not significantly different from controls or not correlated with strength at follow-up, suggesting other mechanisms at play [32]. While mitochondrial content was not significantly reduced [32], a recent study using a septic mouse model has shown that abnormal morphological features in both intermyofibrillar and subsarcolemmal mitochondria are present two weeks following sepsis [186], suggesting that structural changes may be present despite no differences in content. It is unclear if these changes would continue to persist when weakness is still observed.

In those with sustained ICUAW, the reduced capacity of muscle to regenerate as a result of the catabolic damage attained in the ICU likely plays a large role in the observed long-term muscle wasting [68]. This is further supported by dos Santos et al., who found that satellite cell count was decreased at 6 months post-discharge [32]. Alterations in satellite cells are also evident in septic mice, where not only decreased satellite cell count has been reported, but those that remain are largely dysfunctional, with impaired activation and proliferation [187]. Interventions targeting satellite cell generation and activation may therefore assist in minimizing the atrophy present following ICU discharge. Intramuscular injection of mesenchymal stem cells, thought to have a protective effect on satellite cells, has been shown to improve mitochondrial function and may have the potential to improve muscle function in septic mice [187]. In addition, blood flow restricted exercise offers the potential to mediate muscle hypertrophy through protein signaling and satellite cell proliferation to a greater extent than resistance exercise alone [188,189,190] and may be a useful intervention targeting these mechanisms thought to affect sustained ICUAW.

Despite alterations in regenerative capacity, when comparing transcriptional responses of the muscle at 7 days and 6 months post-ICU discharge, it seems that over time the body may attempt to repair muscle by upregulating genes and regulatory factors involved in extra-cellular membrane deposition, contractile function, and muscle structure development [191]. Increased expression of these genes, however, is negatively associated with muscle strength, suggesting abnormal muscle repair. Specifically, genes that share a 5′ UTR binding site for TEAD1—a transcription factor involved in skeletal muscle development [192,193]—are prominently expressed and uniquely upregulated in sustained ICUAW [191]. TEAD1 is known to regulate genes involved in promoting skeletal muscle growth and development such as MAPK, mTOR, and insulin signaling [192]. Phenotypically, increase in the expression of genes involved in extra-cellular membrane reposition is associated with increased collagen deposition in muscle biopsies from survivors of ICU care—possibly hampering muscle repair [191]. In the future, inhibiting fibrosis may enhance regenerative capacity. Accordingly, AMPK has been proposed as a potential target that is involved in the switch towards catabolic metabolism and has implications for pathological fibrosis as well [68].

While less is currently known about the mechanisms responsible for sustained ICUAW, it lends itself to a large area of further exploration needed in the understanding of ICUAW and its unique qualities, as well as how to improve ICUAW through rehabilitation following ICU stay.

## 5. Knowledge Gaps and Avenues to New Insights

### 5.1. Knowledge Gaps

While our understanding of the unique etiology and pathophysiology associated with ICUAW has advanced in recent years, knowledge gaps persist. Namely, what are the mechanistic origins of ICUAW; and are there interventions that can minimize or prevent the deleterious effects of critical illness on muscle tissue? While risk factors for the development of ICUAW are described, it remains difficult to predict which of the 40% of patients who survive their critical illness, and are weak at discharge [194], will continue to exhibit diminished strength 6-months later, and hence, experience sustained disability. As a result, relatively little work has been done to investigate therapeutic interventions that target the ICUAW patient population once back in their community. Of those interventions originally deemed efficacious at improving outcomes in the ICU, some have proven deleterious when tested on a larger patient cohort; thus, highlighting the importance of performing large clinical trials, and the opportunity to improve outcomes if a personalized medicine approach were available. The following section describes opportunities to fill these knowledge gaps, while paving the way for a personalized medicine approach to ICUAW diagnosis and care.

### 5.2. Circulating Factors as Biomarkers and Drivers of ICUAW

Skeletal muscle atrophy and weakness is a common comorbidity with many systemic pathologies including critical illness, diabetes, cancers, sepsis, and muscle disuse [195]. The manifestation of skeletal muscle atrophy is itself clinically significant as skeletal muscle atrophy and weakness is associated with a reduced quality of life, long-term functional disability, and decreased patient survival [196]. Therefore, new biomarkers of muscle mass and function that can be used in routine clinical practice are essential for early diagnosis, prognosis, and disease monitoring.

Blood serum is an easily accessible, and informative component of blood that is constantly circulating through every organ system in our body, including skeletal muscle, and transporting factors that are secreted or excreted under conditions of homeostasis, stress, or different pathologies [197]. Therefore, investigating serum-related biomarkers may shed light on how factors in the blood may drive temporary or permanent muscle atrophy and weakness during and after critical illness. There are many potential biomarkers of skeletal muscle damage, however a caveat of general markers is that they lack disease specificity. For example, levels of CK are elevated in the blood when muscle undergoes damage, however these levels can also fluctuate based on disease, exercise, amount of muscle mass, age, and ethnicity [198,199,200]. Given the variability in circulating CK levels, and more importantly, inability of CK to identify muscle disease or injury subtypes, CK does not prove useful as a potential biomarker for CIM.

Increasing research is being conducted to identify disease-specific circulating biomarkers for those with ICUAW. For example, the growth and differentiation factor 15 (GDF-15), a stress responsive cytokine of the transforming growth factor beta (TGF beta) family [201], has been shown to be elevated in the blood plasma of cardiothoracic ICU patients who developed muscle wasting [202], or were diagnosed with ICUAW [203], and was in the blood serum of critically ill patients with sepsis [204]. Elevated GDF-15 levels were also associated with muscle wasting [202,203], and in the reduced expression of microRNAs that play a role in the regulation of muscle differentiation and recovery [203]. Further, GDF-15 levels at admittance were predictive of ICU survival [204]. Consistently, treatment with GDF-15 in vitro led to elevated expression of ICUAW-associated genes, including muscle atrophy-related ubiquitin ligases, MuRF1 and atrogin1 [203]. This suggests that GDF-15 may be a mediator of muscle atrophy post-operatively and in the cardiothoracic ICU.

In a recent study, levels of GDF-15 detected in blood plasma increased with time over a 7 day period of ICU stay, alongside a simultaneous and significant decline in muscle strength score and CSA of the rectus femoris for those with ICUAW [205]; this trend was not seen in the non-ICUAW group [205]. Since GDF-15 appears to play a role in numerous other pathological conditions, such as cancer and sarcopenia [206], further investigation of the GDF-15 influence in different cell types and disease conditions is warranted. Importantly, these findings offer proof-of-principle that blood-borne mediators may act as biomarkers and drug targets for new therapeutic interventions. Therefore, by incorporating human serum or plasma into 3D human skeletal muscle models, as described below, there may be an opportunity to elucidate molecular mechanisms of ICU-induced muscle atrophy and weakness. Enabling technologies that accurately monitor biomarkers in situ, like the “muscle on a chip” established by Ortega and colleagues [207], offers a synergistic strategy to follow the expression of circulating factors of interest over time.

Given the limitations of pre-clinical models, strategies to conduct discovery research in humans (e.g., critically ill individuals with and without ICUAW in both acute and sustained phases) are essential if we are to identify novel biomarkers of diagnosis, prognosis, and response to therapy. Although in vitro studies do not necessarily reflect the in vivo complexity or an organism, these pre-clinical experiments play an essential role in advancing mechanistic understanding.

### 5.3. 3D Human Skeletal Muscle Models

Animal models afford the opportunity to study disease pathogenesis in the context of a whole organism [208]; however, their use is limited by high costs [209] and ethical considerations. These limitations are compounded when prolonged longitudinal assessment is required, such as with sustained ICUAW. Furthermore, interspecies differences [210] introduce a barrier in translating animal-based studies to the clinic. Human myogenic progenitor cell culture provides an attractive intermediate between animal studies and clinical trials by providing a comparatively inexpensive and species-specific strategy to deconstruct the tissue of interest, in this case skeletal muscle, into modular units (muscle fibers, immune components, fibroblasts, etc.) that are amenable to iterative recombination for cellular and molecular analysis. The simplicity of investigations in a two-dimensional (2D) tissue culture plastic context lends to the domination of this approach for skeletal muscle drug screening efforts [211] and mechanism studies. Although differentiating human myogenic progenitors into multinucleated myotubes is straightforward in 2D culture, the cultures fail to mimic the natural tissue structure of adult human skeletal muscle tissue and lack the cell–cell and cell–extracellular environment interactions that exist in vivo [212,213]. Clever physical [214], topographical [215,216], and chemical [217] cues can be introduced into 2D culture systems to promote myofilament alignment, i.e., fibers extending parallel to the muscle’s force-generating axis, which is critical for skeletal muscle contractility [218]. However, contractile apparatus maturation in 2D culture is a double-edged sword as contraction generally results in myotube breakage or delamination from the dish [219], thereby limiting culture duration and assessments of muscle function.

3D cell culture systems are emerging as a valuable half-way point to bridge the gaps between conventional 2D cell culture systems, whole animal models and clinical trials by allowing the modelling of more physiologically and pathologically relevant processes ex vivo in the context of human cells. The general approach employed to engineer a 3D skeletal muscle culture begins with the encapsulation of skeletal muscle progenitor cells (e.g., derived from biopsy +/- immortalization, pluripotent stem cell derived) within a extracellular matrix-based biomaterial that is deposited into a channel within a fabricated device harboring attachment points at either end, that mimic tendon like attachments [219,220,221,222,223,224]. The tissue self-organization process is guided by the uniaxial tension built between the tendon-like attachments, and drives the formation of a compact tissue containing aligned, multinucleated myotubes that resemble the organization of skeletal muscle tissue. Muscle cells in 3D skeletal muscle culture systems have greater structural maturity than those present in typical 2D cultures, as evidenced by higher expression of the adult MyHC subtypes [219,222], together with expression and function of ryanodine receptors (RyR) and sarco/endoplasmic reticulum Ca^2+^-ATPase (SERCA) channels [224,225], thereby supporting studies of in vivo like Ca^2+^ handling [224]. Furthermore, the cultures can be maintained for several weeks.

Therefore, it is now possible to assess most of the known molecular and functional hallmarks of ICUAW using 3D human skeletal muscle culture models. For example, transduction of skeletal muscle myoblasts with a Ca^2+^ reporter such as GCaMP6 [219,222,224,226], facilitates real time monitoring of spontaneous or induced (chemical or electrical) Ca^2+^ transients [219,222,224] to assess alterations in Ca^2+^ handling. Electrical inactivation and significant decreases in specific force are key hallmarks of ICUAW. In this regard, tissue strength can be measured as an endpoint assay by attaching engineered human muscle tissues to a force transducer [224], or can be repeatedly assayed in the same tissue over time in devices with anchoring posts constructed from a flexible material where tissue-mediated post-deflection captured in short videos is converted to contractile force values [220,221,222], and in conjunction with tissue CSA, is used to calculate specific force. Electrophysiological recordings of myotubes within the 3D tissues offers a potent strategy to analyze membrane electrical activity [227]. These functional metrics integrated with standard cell and molecular biology approaches offers a cadre of ICUAW-relevant readouts to assess downstream of iterative studies designed to uncover combinations of patient and ICU parameters giving rise to ICUAW-like features, and to test possible treatments to prevent or reverse ICUAW. An attractive feature of this approach is the possibility of producing arrays of genetically identical microtissues from a single muscle tissue sample from a healthy human donor [222]. This in turn allows for an evaluation of the influence of sera collected from large cohorts of ICU patients on biologically similar muscle tissues where technical replicates are possible and parameters such as timing and dose can be explored. In this way, 3D skeletal muscle culture systems are positioned to offer a tractable “human model” of ICUAW.

## 6. Conclusions

In the past several years, research into ICUAW has provided great insight into the clinical risk factors and the specific electrophysiological and histological hallmarks that characterize its progression. Important critical insight into the complexity and heterogeneity of the response to injury and repair have required the application of multidisciplinary translational knowledge using human, animal and in vitro studies to elucidate both the molecular mechanisms and the relative importance of different therapeutic strategies in the treatment of early, late and sustained ICUAW. Exploring circulating factors unique to ICUAW patients in the context of tissue engineered culture models of human skeletal muscle may bring us one step closer to resolving the long-standing debate as to whether the origin of ICUAW is a result of the critical illness, co-morbidities, muscle unloading, or ICU treatments, a systemic reaction circulating within the body, or combinations therein. Furthermore, the availability of a culture model of ICUAW could facilitate in expediting the diagnosis of ICUAW and fast track the discovery of putative treatments.

## Figures and Tables

**Table 1:** Summary of Existing Intensive Care Unit Animal Models and Their Key Characteristics.

ICU Model	Strain	Sex	Age	ICUAW Interventions and Triggers	Duration of Mechanical Ventilation	ICUAW Hallmarks Replicated	Affected Muscle Groups
**Porcine**	*Sus scrofa* [46]	F [46,47,48,49,50]	Piglets	Neuromuscular blocking agent [47,48,49]	27 h [51]	Reduced force generation [47,50,52]	Diaphragm [52]
				Mechanical ventilation [47,48,49,50,51,52,53]	48 h [53]	Decreased muscle membrane excitability [47,48,51,53]	Limb [47,48,49,50,51,52,53]
				Corticosteroids [47,48,49]	72 h [52]		Masseter [48,49]
				Sepsis [47,48,49,50,51,52,53]	5 days [46,47,49,50]		
				All * [47,48]			
**Rat**	Sprague- Dawley [54,55,56,57,58]	F [54,56,58]	-	Neuromuscular blocking agent [54,55,56,57,58]	0–≥14 days [54,55,56,57,58]	Muscle Atrophy [54,55,56,57,58]	Diaphragm [54,57]
				Mechanical ventilation [54,55,56,57,58]		Preferential loss of myosin [54,56,57,58]	Limb [55,56,57,58]
						Reduced force generation [54,56,58]	Masseter [57]

* All includes neuromuscular blocking agents + mechanical ventilation + corticosteroids + sepsis. ICUAW triggers and interventions listed are in addition to immobilization induced by sedation/anesthesia.

## Abbreviations

2D	2- Dimensional
3D	3- Dimensional
AKT	Protein kinase B
AMPK	5′ Adenosine monophosphate - activated protein kinase
ATP	Adenosine triphosphate
BGP-15	Poly (adenosine 5′-diphosphate)–ribose] polymerase 1 (PARP-1) Inhibitor
CIM	Critical illness myopathy
CIP	Critical illness polyneuropathy
CK	Creatine kinase
CMAP	Compound muscle action potential
COVID-19	Coronavirus disease 2019
CSA	Cross sectional area
FBOX31,	F-box only protein 31
FDA	Food and drug administration
GCaMP6	Genetically encoded calcium indicator
GDF-15	Growth and differentiation factor 15
HSP	Heat shock protein
ICU	Intensive care unit
ICUAW	ICU - acquired weakness
IF4E- binding protein 1	Eukaryotic translation initiation factor 4E binding protein 1
IGF1	Insulin-like growth factor -1
IL-1	Interleukin-1
IL-6	Interleukin-6
LC3 -I/II	Microtubule-associated protein light chain 3 (I/II)
MAFbx	Muscle-atrophy F box protein
MAPK	Mitogen-activated protein kinase
MRC	Medical research council
mRNA	Messenger RNA
mTOR	Mammalian target of rapamycin
mTORC1	Mammalian target of rapamycin complex 1
mTORC2	Mammalian target of rapamycin complex 2
MuRF1	Muscle ring finger protein 1
MuRF2	Muscle ring finger protein 2
MV	Mechanical ventilation
MyHC	Myosin heavy chain
NF-kB	Nuclear factor-κB
NMBA	Neuromuscular blocking agent
PGC-1	Peroxisome proliferator-activated receptor gamma coactivator 1-alpha
PI3K	Phosphoinositide 3-kinase
RNA	Ribonucleic acid
RyR	Ryanodine receptors
SARS-CoV-2	Severe acute respiratory syndrome coronavirus 2
SERCA	Sarco/endoplasmic reticulum Ca2+ -ATPase
TEAD1	TEA domain transcription factor 1
TFAM	Mitochondrial transcription factor A
TGFβ	Transforming growth factor β
TNFα	Tumor necrosis factor α
TRIM32	Tripartite motif-containing protein 32
UNC-45B	Unc-45 myosin chaperone B
UPS	Ubiquitin proteasome system
UTR	Untranslated region
